# Comparative Genome Analysis of Illumina, Nanopore, and Hybrid Approaches: A Case Study of the Aquaculture Isolate 160P

**DOI:** 10.3390/pathogens15030293

**Published:** 2026-03-06

**Authors:** Izzet Burcin Saticioglu, Janset Bozkurt, Muhammed Duman

**Affiliations:** 1 Department of Aquatic Animal Diseases, Faculty of Veterinary Medicine, Bursa Uludag University, 16059 Bursa, Türkiye; mduman@uludag.edu.tr; 2 Institute of Aquaculture, Faculty of Natural Sciences, University of Stirling, Stirling FK9 4LA, UK; 3 Department of Molecular Biology and Genetics, Faculty of Arts and Sciences, Bursa Uludag University, 16059 Bursa, Türkiye; bozkurtjanset@gmail.com

**Keywords:** *Aeromonas*, hybrid genome assembly, Oxford Nanopore Technologies

## Abstract

In this study, we comparatively assessed short-read (Illumina), long-read (Oxford Nanopore Technologies, ONT), and hybrid (Illumina + ONT) sequencing strategies for bacterial genome analysis using the aquaculture-derived isolate 160P. Genomic DNA was extracted and sequenced on Illumina paired-end and ONT long-read platforms, and de novo assemblies were generated using SPAdes, Canu, Flye, and Unicycler under short-read-only, long-read-only, and hybrid workflows, followed by evaluation with QUAST assembly metrics. Among the tested approaches, the hybrid Unicycler assembly provided the highest contiguity, yielding seven contigs and a dominant 4.55 Mb contig consistent with near-complete chromosomal representation. Downstream analyses included functional genome annotation and in silico screening of antimicrobial resistance determinants (CARD), virulence-associated genes (VFDB), and secondary metabolite biosynthetic gene clusters (antiSMASH). Comparative genomic relatedness based on Average Nucleotide Identity (ANI) and digital DNA–DNA Hybridization (dDDH) indicated that 160P is most closely related to *Aeromonas sobria* CECT 4245^T^ yet falls below commonly applied species-level thresholds, supporting its placement as a genomically distinct lineage warranting further taxonomic investigation. Collectively, these findings underscore the value of hybrid sequencing for improving assembly continuity, enhancing annotation completeness, and strengthening taxonomic resolution in bacterial pathogen genomics.

## 1. Introduction

Accurate bacterial genome assembly is essential for high-resolution taxonomic identification, structural genome characterization, and comparative microbial genomics. With the growing need for complete, contiguous genome sequences, especially for novel or uncultured strains, sequencing technologies have become critical tools in modern microbiology. Short-read sequencing platforms, such as those developed by Illumina, are known for their high base accuracy, cost-effectiveness, and robust downstream analysis pipelines. However, their limited read length often results in fragmented assemblies, especially in regions containing repeats, insertion sequences, or complex structural variations [1,2]. On the other hand, long-read sequencing platforms such as those from Oxford Nanopore Technologies (ONT) produce reads that span repetitive or structurally challenging genomic regions, significantly improving assembly contiguity. Nevertheless, these long-read platforms suffer from higher base error rates, which can compromise assembly accuracy and downstream annotation [2]. To address the limitations of both technologies, hybrid sequencing strategies that integrate short-read and long-read data have gained considerable traction. These approaches aim to leverage the accuracy of Illumina reads with the structural resolution of Nanopore reads, resulting in more accurate and contiguous assemblies [3]. However, hybrid assembly is not universally superior. Recent studies have demonstrated that high-quality long-read platforms, including PacBio HiFi and optimized ONT workflows, can independently generate complete and highly accurate bacterial genomes without hybrid scaffolding [3,4,5]. Furthermore, hybrid graph integration may propagate ambiguities originating from short-read assembly graphs, potentially introducing structural inconsistencies [3,5]. Therefore, the optimal assembly strategy should be selected based on sequencing quality, genome complexity, study objectives, and resource considerations, particularly in epidemiological contexts.

Moreover, the quality of genome assemblies directly influences genome-based taxonomic inference, particularly when evaluated using Average Nucleotide Identity (ANI) and digital DNA–DNA hybridization (dDDH), which are widely accepted metrics for prokaryotic species delineation, with commonly applied thresholds of approximately 95–96% for ANI and ~70% for dDDH.

In this study, we performed a comparative genome assembly analysis of the bacterial isolate 160P using three sequencing approaches: short-read (Illumina), long-read (Nanopore), and a hybrid strategy combining both. The primary objective was to determine whether hybrid sequencing improves assembly contiguity and enhances the reliability of downstream genomic analyses compared with single-technology approaches. The resulting assemblies were systematically evaluated using quality and completeness metrics, including total genome length, number of contigs, N50, L50, GC content, gene count, and recovery of rRNA and tRNA genes. Furthermore, we assessed how differences in assembly quality influence genome-based taxonomic inference using ANI and dDDH analyses, thereby examining the impact of sequencing strategy on species-level classification accuracy.

## 2. Materials and Methods

### 2.1. Bacterial Isolate and DNA Extraction

The bacterial isolate 160P was obtained from the culture collection of the Department of Aquatic Animal Diseases, Faculty of Veterinary Medicine, Bursa Uludağ University (Türkiye). The isolate was originally recovered in Bursa (Türkiye) in 2021 from an aquarium fish (*Pristolepis fasciata*) and stored at –80 °C in Tryptic Soy Broth (TSB, Merck, Darmstadt, Germany) broth supplemented with 20% glycerol until further use. Prior to sequencing, the isolate was revived and routinely propagated on Tryptic Soy Agar (TSA, Merck, Darmstadt, Germany) at 28 °C for 24 h before downstream analyses. Genomic DNA was successfully extracted from the revived 160P isolate. DNA purity was assessed using a Multiskan™ GO Microplate Reader (Thermo Fisher Scientific, Waltham, MA, USA), yielding an A260/280 absorbance ratio of 2.049. DNA concentration was further quantified using a Qubit™ 4.0 Fluorometer (Invitrogen, Thermo Fisher Scientific, Waltham, MA, USA) which determined a double-stranded DNA (dsDNA) concentration of 87.50 ng. The obtained DNA quantity and purity were considered sufficient for both Illumina library preparation and Oxford Nanopore Technologies (ONT) ligation-based library preparation, meeting the minimum input requirements for downstream sequencing.

### 2.2. Library Preparation and Sequencing

For Illumina sequencing, genomic DNA was used for library preparation with the Illumina DNA Prep kit following the manufacturer’s instructions. Libraries were generated via enzymatic fragmentation, yielding an expected insert size distribution of approximately 300–500 bp according to the manufacturer’s specifications. After adapter ligation and indexing with Illumina UD index adapters, libraries were PCR-enriched and quantified using a Qubit™ 4 Fluorometer. Sequencing was performed in paired-end mode (2 × 150 bp) on an Illumina NovaSeq 6000 platform Illumina NovaSeq 6000 platform (Illumina, San Diego, CA, USA), generating approximately 3 Gbp of raw data corresponding to an estimated genome coverage depth of ~650×. Adapter trimming and quality filtering were performed prior to assembly using the Bacterial and Viral Bioinformatics Resource Center (BV-BRC) Fastq Utilities pipeline.

Long-read sequencing was performed on an ONT PromethION 2 Solo platform (Oxford Nanopore Technologies, Oxford, UK) using an R10.4.1 flow cell (FLO-PRO114M). Libraries were prepared using the Kit 14 native barcoding ligation workflow (SQK-NBD114.24). The sequencing run was conducted for 24 h under MinKNOW control (Version 25.09.16). Basecalling was performed using a minimum quality score cutoff of Q ≥ 9. No adaptive sampling was applied during the sequencing run. Reads passing the quality threshold were used for downstream assembly without additional length-based filtering.

Raw FASTQ files generated by the Illumina and ONT platforms were uploaded to the BV-BRC web interface for downstream processing [6]. Read quality was evaluated using Fastq Utilities (BV-BRC), including GC content, read-length distribution, and ambiguous base content (N), and trimming/quality filtering were applied as required.

### 2.3. Bioinformatic Analyses

De novo genome assemblies were generated separately for Illumina-only, ONT-only, and hybrid datasets using SPAdes (version 4.0.0) [7], Canu (Version 2.0) [1], Flye (version 2.9.1-b1780) [8], and Unicycler (version 0.4.8) [2] through the Genome Assembly service of BV-BRC [6]. Assemblies were performed using the standard parameters implemented within the BV-BRC pipeline to ensure methodological consistency and comparability across sequencing strategies. Prior to assembly, Illumina reads were adapter- and quality-trimmed and normalized to a target depth of 150×, while ONT reads were used following platform basecalling. Contigs shorter than 1000 bp or with coverage below 5× were excluded from the BV-BRC workflow. For long-read assemblies generated with Canu, two rounds of Racon polishing were applied. ONT-only assemblies generated with Flye were subjected to the consensus refinement procedures implemented within the BV-BRC pipeline. Illumina-based assemblies generated with SPAdes were polished using one round of Pilon, while hybrid assemblies produced with Unicycler incorporated internal polishing steps integrating short- and long-read correction. Average sequencing depth was calculated for both Illumina and ONT datasets to assess read support and assembly robustness. Assembly quality and integrity were evaluated using a multi-tiered approach. Contiguity and structural accuracy were assessed with QUAST (v5.3.0) [9], including N50, L50, and misassembly statistics (relocations, translocations, and inversions). Genomic completeness and contamination were determined using CheckM2 (v1.1.0) [10], and results were cross-validated with BUSCO (v6.0.0) [11], against the bacteria_odb10 database to confirm recovery of universal single-copy orthologs and overall assembly completeness.

The most complete and structurally consistent hybrid assembly was selected for downstream analyses. Taxonomic placement was initially assessed using EzBioCloud 16S rRNA similarity searches [12]. Species-level relatedness was evaluated using average nucleotide identity (ANI) calculated with JSpeciesWS (version 5.0.3) [13] and digital DNA–DNA hybridization (dDDH) analysis performed via the Type (Strain) Genome Server (TYGS) [14]. Within TYGS, pairwise intergenomic distances were computed using the Genome BLAST Distance Phylogeny (GBDP) method under recommended settings, and dDDH values with confidence intervals were obtained through GGDC 4.0. Phylogenomic relationships were inferred based on GBDP distances using FASTME (Version 2.0) with 100 bootstrap replicates, and the resulting tree was midpoint-rooted.

Species-level relatedness was evaluated using digital DNA–DNA hybridization (dDDH) via the TYGS platform (GGDC formula d4) and average nucleotide identity calculated with JSpeciesWS [13], applying both ANIb (BLAST-based) and ANIm (MUMmer-based) algorithms. Comparisons were performed against the closest *Aeromonas* type strain genomes automatically identified by TYGS.

Functional annotation of the finalized assembly was performed using the BV-BRC Comprehensive Genome Analysis service [6]. Antimicrobial resistance determinants were identified using the Comprehensive Antibiotic Resistance Database (CARD) via the Resistance Gene Identifier (RGI, Version 6.0.5) under the “Perfect” and “Strict” hit criteria. A minimum sequence identity threshold of 80% and a minimum coverage cutoff of ≥80% of the reference gene length were applied to retain high confidence matches [15]. Virulence-associated genes were screened using the Virulence Factor Database (VFDB) through VFanalyzer [16]. Biosynthetic gene clusters were predicted using antiSMASH v8.0.4 with “relaxed” detection settings [17].

## 3. Results

### 3.1. Raw Read Assessment

A total of 20,795,556 Illumina paired-end reads were generated (10,397,778 reads in each of R1 and R2), with read lengths ranging from 35 to 151 bp. The total base yield per file was approximately 1.5 Gbp, resulting in an estimated genome coverage depth of ~650× based on the final assembly size. The mean GC content was 55%, consistent with the assembled genome. Read-quality profiling demonstrated high base-call accuracy across read positions, with average Phred quality scores ranging between Q34 and Q36.

For ONT sequencing, a total of 70,079 reads were generated, yielding 180,223,737 bp (~180 Mb) of sequence data. The mean read length was 2571 bp, with a median read length of 999 bp and a read N50 of 6655 bp, corresponding to an estimated coverage depth of approximately 39× based on the final genome size (~4.57 Mb). The mean GC content was 57.0%. Per-read quality profiling indicated that Phred-equivalent scores were centered around Q29 (mean Q ≈ 28.0; interquartile range Q25–Q31), and 94.2% of reads exhibited a mean quality score ≥ Q20. In the module summary, 20% of quality-control modules were reported as failed, primarily reflecting read-length distribution characteristics typical of long-read sequencing datasets. Specifically, failures were associated with the “Per base sequence quality”, “Per sequence GC content”, and “Sequence length distribution” modules, which are commonly flagged in ONT data due to broader read-length variability and platform-specific quality score distributions. These patterns are expected for long-read technologies and did not compromise downstream genome assembly, as sufficient read quality (Q ≥ 9 threshold) was achieved.

### 3.2. Assembly Results

**Figure 1:**
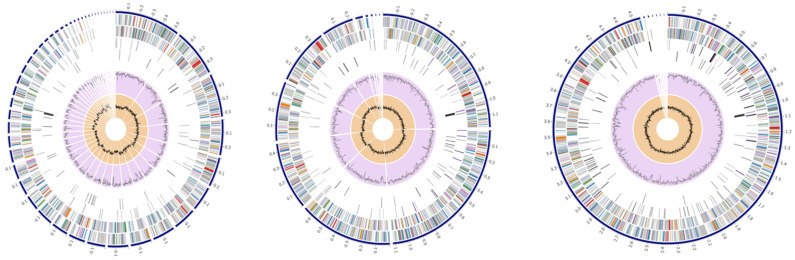
Circular genome maps of the 160P isolate based on Illumina (**left**), ONT (**middle**), and hybrid (**right**) assemblies. From outer to inner rings, the circles represent: contigs, coding sequences (CDSs) on the forward strand, CDSs on the reverse strand, RNA genes (rRNA and tRNA), CDSs with homology to known antimicrobial resistance genes, CDSs with homology to known virulence factors, GC content, and GC skew.

The comparison of genome assemblies derived from ONT, Illumina, and hybrid sequencing datasets revealed significant differences in contiguity and structural resolution (Table 1). Among the single-technology approaches, the ONT-only assembly by Flye yielded a highly contiguous draft with 13 contigs (4,517,234 bp) and an N50 of 1,116,123 bp (L50 = 2). In contrast, the Illumina-only assembly generated by Unicycler exhibited higher fragmentation, resulting in 47 contigs (4,454,205 bp) with a substantially lower N50 of 182,099 bp (L50 = 8). The hybrid strategy demonstrated the most superior performance; the Unicycler hybrid assembly achieved near chromosome-scale reconstruction, comprising only 7 contigs (4,575,085 bp) with an N50 of 4,552,216 bp (L50 = 1). Sequencing depths reached 209× for Illumina and 38× for ONT datasets, providing robust coverage for reliable consensus generation (Figure 1). Structural validation via QUAST (v5.3.0) confirmed the accuracy of the reconstructed genomes, as no substantial large-scale misassemblies, such as relocations, translocations, or inversions, were detected across any of the strategies.

Biological and statistical quality assessments further highlighted the exceptional purity and completeness of all assemblies. CheckM2 analysis indicated that contamination levels were remarkably low: 0.16% for the hybrid, 0.17% for the Illumina-only, and 0.30% for the ONT-only assemblies. Furthermore, BUSCO analysis (v6.0.0; bacteria_odb10 dataset, *n* = 124) achieved 100% completeness for all assemblies, with 122 single-copy and 2 duplicated orthologs identified and no fragmented or missing genes. The high degree of concordance between contiguity metrics, structural validation, and universal gene recovery confirms the high-fidelity reconstruction of the *Aeromonas* sp. 160P genome, with the hybrid approach providing the most contiguous and biologically representative assembly. The Illumina and ONT sequencing datasets have been deposited in the NCBI Sequence Read Archive (SRA) under accession numbers SRR37315471 and SRR37315472, respectively, within BioProject PRJNA1420202. The final hybrid genome assembly has been deposited in GenBank under accession number JBUXKV000000000, with the corresponding GenBank Assembly accession number GCA_055193095.

### 3.3. Comparative Bioinformatic Analyses of Illumina, ONT, and Hybrid Assemblies

**Figure 2:**
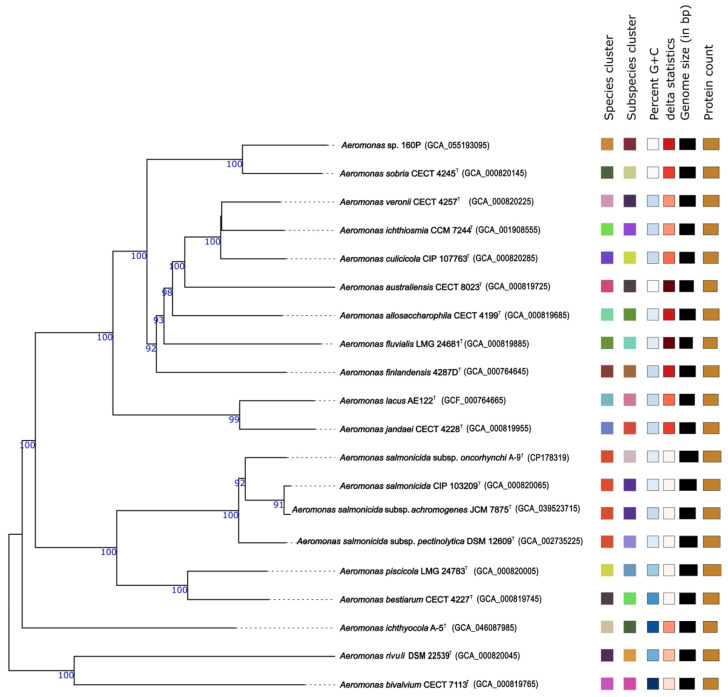
Genome-based phylogenomic tree of isolate 160P inferred using TYGS. GBDP distances (formula d5) were used to reconstruct the tree with FastME (100 bootstrap replicates). Branch lengths reflect GBDP distance, and values above branches indicate bootstrap support (>60%). The tree was midpoint-rooted.

The Illumina–Unicycler assembly of isolate 160P comprised 47 contigs totaling 4.45 Mb, with an N50 of 182,099 bp. Functional annotation predicted 4174 protein-coding sequences (CDSs), 100 tRNAs, and three rRNA genes. Given the repetitive nature of rRNA operons in *Aeromonas* genomes, the recovery of a single complete operon likely reflects repeat collapse associated with short-read assembly rather than true copy number reduction, as multiple rRNA copies were resolved in the ONT and hybrid assemblies. The antimicrobial resistance profile included *dfrB1* (trimethoprim), *qacEΔ1* (disinfectant tolerance), *OXA-959*, *aadA*, *catB2*, *sul1*, and *cphA3* hits. Virulence-associated features included Flp/Tad pili, the Msh pilus, type I fimbriae, and type II, III, and VI secretion systems (T2SS, T3SS, and T6SS). Secondary metabolite analysis identified RiPP-like, NRP-metallophore, terpene, and homoserine lactone–associated clusters. Only three rRNA genes were detected, and the LPS *rfb* locus was not identified in this assembly. Genome-relatedness analyses were performed against the closest *Aeromonas* type strain genomes identified via TYGS, including *A. sobria* CECT 4245^T^ (assembly accession: GCA_000820145). For the Illumina assembly, ANIb and ANIm values were 94.80% and 95.18%, respectively, while the digital DNA–DNA hybridization value (dDDH; GGDC formula d4) was 60.5% (Table 2). To further evaluate the taxonomic placement of isolate 160P, a genome-based phylogenomic analysis was conducted using the TYGS. Pairwise intergenomic distances were calculated using the Genome BLAST Distance Phylogeny (GBDP) approach, and a phylogenetic tree was inferred with FASTME based on formula d5 distances (Figure 2). The resulting tree comprised 17 species clusters and 19 subspecies clusters. Isolate 160P formed a well-supported lineage within the *A. sobria* species cluster, grouping most closely with the type strain *A. sobria* CECT 4245^T^. Bootstrap support values were high (average branch support 95.3%), indicating robust phylogenomic placement. Although ANI and dDDH values fell slightly below conventional species thresholds, the genome-based tree supports close affiliation of isolate 160P with the *A. sobria* lineage rather than placement in a clearly distinct species-level clade.

The ONT–Flye assembly yielded 13 contigs and improved contiguity (N50 ≈ 1.1 Mb). Annotation predicted 4207 CDSs, 131 tRNAs, and 30 rRNA genes. The AMR and virulence profiles were broadly concordant with those observed in the Illumina assembly. Four secondary metabolite clusters were detected, whereas the LPS *rfb* locus was again not identified. ANI (ANIb = 94.95%, ANIm = 95.17%) and dDDH (60.7%) values were consistent with those obtained for the Illumina assembly when compared against the same *A. sobria* CECT 4245^T^ type strain genome, remaining below established species delineation thresholds (95–96% ANI; 70% dDDH) (Table 2).

The hybrid–Unicycler assembly achieved the highest overall assembly quality, producing seven contigs with a total genome size of 4.57 Mb and an N50 of 4.55 Mb. Annotation predicted 4246 CDSs, 131 tRNAs, 30 rRNA genes, and 89 repeat regions. The AMR gene repertoire was consistent across assemblies; however, the LPS *rfb* locus was detected only in the hybrid assembly, suggesting improved reconstruction of this genomic region. ANI (ANIb = 94.91%, ANIm = 95.18%) and dDDH (60.7%) values again indicated closest genomic similarity to *A. sobria* CECT 4245^T^ while remaining below species-level thresholds. Collectively, genome-based metrics obtained from independent assemblies consistently support the placement of isolate 160P as most closely related to *A. sobria*, yet below accepted species delineation criteria.

## 4. Discussion

In this study, we compared short-read (Illumina), long-read (Oxford Nanopore Technologies, ONT), and hybrid (Illumina + ONT) whole-genome sequencing strategies for the aquaculture-derived bacterial isolate 160P. Across all pipelines, hybrid sequencing produced the most contiguous assembly and the most complete downstream annotation, whereas Illumina-only assemblies were the most fragmented and ONT-only assemblies showed intermediate contiguity. Importantly, improved contiguity was associated with more consistent recovery of multi-copy features (such as rRNA genes), repeat-associated regions, and biologically relevant loci (including LPS-associated regions), supporting previous reports that assembly continuity can influence downstream genomic interpretation and locus-level inference [2,3].

Next-generation sequencing has made bacterial whole-genome sequencing a practical routine in many laboratories, enabling genome-wide characterization for species assignment, outbreak investigation, AMR surveillance, and exploration of virulence/metabolic potential beyond marker genes [18]. However, the extent to which these goals are achieved depends strongly on assembly completeness and correctness, because accessory genes, mobile elements, and multi-copy loci are often embedded in repetitive or structurally complex regions that draft assemblies may fragment or misrepresent. In our study, this consideration was central: we used isolate 160P to evaluate how commonly used sequencing strategies affect assembly contiguity and downstream interpretability. The patterns we observed support the general view that sequencing choice is not merely a technical preference but a determinant of how confidently genomic context (operon structure, island boundaries, and locus-level organization, etc.) can be interpreted.

A consistent finding across microbial genomics is that platform-specific error profiles and read-length distributions drive different assembly outcomes. Illumina short reads are typically highly accurate and well supported by mature analytical pipelines, but their limited length often prevents spanning repeats, yielding fragmented assemblies [2]. By contrast, ONT long reads can bridge repeats and improve structural resolution, although systematic error modes can necessitate careful polishing to reach high base-level accuracy and to protect downstream gene prediction and comparative analyses [19]. Our raw datasets reflected the expected platform-specific characteristics: Illumina provided high base-level accuracy with uniform read quality, whereas ONT generated longer reads that improved structural resolution despite broader quality score distributions typical of long-read technologies. In line with the literature, the Illumina-only dataset provided very strong base-level accuracy but limited repeat-bridging capacity, while ONT contributed the long-range continuity needed to connect complex genomic regions; together, these complementary strengths underscore why mixed strategies are often preferred when both sequence accuracy and structural completeness are priorities [6,20].

This trade-off became clear at the assembly level. Benchmarking studies frequently report that short-read-only approaches yield higher contig counts, long-read-only approaches improve contiguity but can retain systematic errors, and hybrid strategies often provide the best balance by using long reads to resolve structure and short reads to correct sequence-level errors [2,20]. At the assembly level, Illumina-only data resulted in the most fragmented genome reconstruction, ONT-only data substantially improved contiguity, and the hybrid strategy achieved near chromosome-scale continuity, providing the most structurally resolved assembly. The key implication is not only improved summary statistics, but also a more interpretable genome: the multi-megabase contig in the hybrid assembly suggests that many repeats and ambiguous graph branches were bridged, reducing uncertainty in gene order and the genomic context of AMR/virulence determinants elements that are frequently central to phenotype and epidemiological inference.

Annotation completeness tracked assembly contiguity. The Illumina– Unicycler assembly recovered 4174 CDSs, 100 tRNAs, and only three rRNA genes, whereas both ONT–Flye and hybrid–Unicycler recovered 30 rRNA genes and 131 tRNA genes, consistent with improved resolution of multi-copy rRNA operons when long-range information is available [17]. The reported 30 rRNA genes represent individual 16S, 23S, and 5S gene predictions rather than 30 complete operons. This number is biologically plausible for a ~4.5 Mb *Aeromonas* genome, which typically contains 8–10 rRNA operons (24–30 rRNA genes in total). The lower count in the Illumina-only assembly likely reflects repeat collapse associated with short-read assembly, whereas the ONT-only and hybrid assemblies resolved these loci in numbers consistent with expected operon multiplicity. The predicted rRNA genes corresponded to full-length annotations rather than fragmented sequences. Repeat-associated features were identified in the hybrid assembly (89 annotated repeat regions) during the RASTtk-based annotation process, whereas no repeat regions were annotated in the Illumina–Unicycler or ONT–Flye assemblies. This difference likely reflects improved assembly contiguity in the hybrid genome, which facilitates the detection of internally repeated sequences that may be fragmented or unresolved in more fragmented draft assemblies.

A major practical implication is that assembly quality influences not only contiguity metrics but also the confidence of biologically meaningful presence/absence calls. In our analysis, core AMR determinants were consistently detected across assemblies, indicating concordant gene-level predictions despite differences in contiguity. The LPS-associated *rfb* locus was identified in the hybrid assembly within a continuous chromosomal region. Under the same screening criteria, comparable high-confidence detection was not observed in the more fragmented assemblies. This difference most likely reflects variation in assembly continuity and locus reconstruction rather than confirmed gene absence. Given the validated structural integrity and completeness of the assemblies, AMR and virulence gene predictions are considered reliable, although loci located in repetitive or structurally complex regions should be interpreted cautiously in draft genomes. Similar considerations apply to virulence-associated and secondary metabolite clusters, where accurate reconstruction of cluster boundaries and synteny may depend on assembly contiguity. These interpretations are supported by validated assembly integrity and cross-platform concordance of gene detection results [16,17].

Our findings align with a broad literature showing that hybrid strategies often provide the best balance between structural completeness and base-level accuracy for bacterial genomes. Unicycler was developed to resolve bacterial assemblies by combining accurate short reads with long reads that bridge repeats and structural complexities [2]. Benchmarking studies in bacterial pathogens have similarly reported that hybrid assemblies typically improve contiguity and reduce assembly artifacts relative to single-technology approaches, with downstream benefits for gene finding and genomic interpretation [21]. More recent benchmarking also supports the conclusion that Illumina offers consistently high raw-read accuracy, ONT improves resolution of repetitive regions (including rRNA operons), and hybrid assemblies provide comprehensive overall results when annotation integrity and complex loci matter [22].

Beyond technical benchmarking, this case study provides a biologically contextualized comparison of sequencing strategies. Rather than focusing solely on contiguity metrics, we demonstrate how assembly structure influences downstream biological interpretation, including recovery of multi-copy operons, repeat-associated loci, and the robustness of genome-based taxonomic inference. By explicitly linking assembly continuity to annotation completeness and comparative genomic reliability in an aquaculture-derived *Aeromonas* isolate, this study extends existing benchmarking literature from a purely technical evaluation toward a functional and taxonomic interpretability perspective.

It is important to acknowledge that hybrid assembly is not universally superior for prokaryoti reconstruction. Recent benchmarking studies have demonstrated that high-fidelity long-read datasets, including PacBio HiFi and optimized ONT workflows, can independently generate complete and highly accurate bacterial genomes without hybrid scaffolding [3,4,5]. In certain contexts, hybrid graph integration may propagate ambiguities originating from short-read assembly graphs, potentially affecting structural accuracy. Moreover, in epidemiological or resource-limited settings, the cost–benefit balance between hybrid sequencing and single-technology approaches is an important consideration. Therefore, the optimal assembly strategy should be selected based on sequencing depth, read accuracy, genome architecture, and specific research objectives rather than assuming a universally superior framework.

Bioinformatics choices further shape how effectively each data type is translated into a reliable genome. For short reads, de Bruijn graph assemblers such as SPAdes typically perform well at high coverage but remain fundamentally limited by repeats longer than the reads [7]. For long reads, assemblers such as Flye leverage repeat-graph concepts tailored to error-prone reads, increasing contiguity but also increasing dependence on polishing to mitigate systematic errors [2,19]. Accordingly, polishing strategies that combine long-read consensus refinement (such as Racon) with short-read correction (like Pilon) are widely used to approach near-finished microbial assemblies [23,24], and recent evaluations emphasize that homopolymer- and repeat-associated errors remain among the most persistent issues—precisely those that can distort gene prediction and comparative analyses [25,26,27]. In our results, downstream annotation signals were consistent with this framework: the Illumina-only assembly recovered markedly fewer rRNA genes (3 rRNA) than the ONT-only and hybrid assemblies (30 rRNA), indicating that multi-copy loci were better resolved when long-range information was available. Moreover, repeat-associated features and complex loci showed improved recovery in the hybrid assembly, supporting the interpretation that hybrid sequencing not only improves contiguity but also restores difficult genomic regions that can be underrepresented in fragmented drafts.

Finally, assembly quality can influence genome-based taxonomy and comparative genomics, though robust distance metrics tend to remain stable when assemblies are sufficiently complete. Species delineation commonly relies on thresholds around ~95–96% ANI and ~70% dDDH, while values below these cutoffs can indicate close relatedness without meeting species-level boundaries [28,29]. In our study, ANI (~94.8–95.2%) and dDDH (~60.5–60.7%) were consistent across Illumina-only, ONT-only, and hybrid assemblies, suggesting that isolate 160P is closely related to *A. sobria* CECT 4245^T^ but may fall below species delimitation thresholds. The agreement across strategies implies that our taxonomic signal is not driven by a single platform artifact; nevertheless, the superior structural completeness of the hybrid assembly provides the most reliable substrate for subsequent fine-scale comparative genomics (e.g., genomic island mapping, operon-level comparisons, and locus-context interpretation) and any future taxonomic resolution work that may depend on high-contiguity references [14]. Importantly, isolate 160P represents a particularly informative model for evaluating assembly strategy because its genome combines borderline species-level similarity with structurally complex features, including multi-copy rRNA operons and repeat-associated regions that are prone to fragmentation in short-read assemblies. These characteristics make 160P well suited to illustrate how assembly contiguity can influence both structural genome reconstruction and the confidence of downstream comparative and taxonomic interpretations. Nevertheless, conclusions drawn from a single isolate should be interpreted cautiously, as assembly performance may vary across taxa with differing repeat content, plasmid architecture, or genome plasticity. Future studies incorporating multiple isolates and sequencing depth gradients would further clarify the generalizability of these findings.

## 5. Conclusions

Illumina-only sequencing generated highly accurate reads but resulted in a fragmented draft genome. ONT long reads substantially improved assembly continuity, whereas the hybrid strategy provided the highest structural completeness and the most reliable genomic context reconstruction. Within the scope of this study, hybrid sequencing offered the most balanced framework for achieving both contiguity and annotation robustness, while recognizing that optimal strategy selection may vary depending on organismal complexity and sequencing depth.

## Figures and Tables

**Table 1:** Assembly statistics for ONT, Illumina, and hybrid datasets using different assemblers.

Data Type	Assembler	Contigs	Total Length (bp)	GC (%)	Longest Contig (bp)	N50 (bp)	L50
ONT	Canu	69	4,992,899	58.07	458,904	178,605	8
ONT	Flye	13	4,517,234	58.13	1,180,313	1,116,123	2
ONT	Unicycler	26	4,266,824	58.08	545,535	264,592	6
Illumina	SPAdes	60	4,449,933	58.05	562,351	158,359	11
Illumina	Unicycler	47	4,454,205	58.07	536,629	182,099	8
Hybrid	Canu	148	5,435,219	58.01	458,909	159,294	10
Hybrid	Flye	18	4,526,748	58.13	1,180,867	1,116,152	2
Hybrid	SPAdes	19	4,502,815	58.05	1,639,518	988,566	2
Hybrid	Unicycler	7	4,575,085	57.97	4,552,216	4,552,216	1

**Table 2:** Comparative Genomic and Functional Annotations of Illumina, ONT, and Hybrid Assemblies.

Feature/Metric	Illumina–Unicycler	ONT–Flye	Hybrid–Unicycler
Contigs	47	13	7
Total Length (bp)	4,454,205	4,517,234	4,575,085
N50 (bp)	182,099	1,116,123	4,552,216
CDS	4174	4207	4246
tRNA	100	131	131
rRNA	3	30	30
AMR Genes	*dfrB1*; *qacEΔ1*; *OXA-959*; *aadA*; *catB2*; *sul1*; *cphA3*; *rsmA*; EF-Tu (R234F).	Same	Same
Virulence Factors	Type IV pili; fimbriae; flagella; secretion systems (T2SS, T3SS, T6SS); toxins.	Same	Same + LPS *rfb* locus
Secondary Metabolite Clusters	RiPP-like; terpene; NRP-metallophore; homoserine lactone (hserlactone).	Same	Same
ANIb/ANIm/dDDH	94.80/95.18/60.5	94.95/95.17/60.7	94.91/95.18/60.7

ANIb, ANIm and dDDH values were calculated against *A. sobria* CECT 4245^T^ (GenBank assembly accession GCA_000820145).

## Data Availability

The original contributions presented in this study are included in the article. The genome assembly generated in this study has been deposited in the NCBI GenBank database, and the corresponding accession numbers are provided in the manuscript. Further inquiries can be directed to the corresponding author.

## Abbreviations

ANI	Average Nucleotide Identity
dDDH	Digital DNA–DNA Hybridization
ONT	Oxford Nanopore Technologies
BV-BRC	Bacterial and Viral Bioinformatics Resource Center
QUAST	Quality Assessment Tool for Genome Assemblies
CARD	Comprehensive Antibiotic Resistance Database
VFDB	Virulence Factor Database
BGC	Biosynthetic Gene Cluster
AMR	Antimicrobial Resistance
PCR	Polymerase Chain Reaction
GC	Guanine–Cytosine
rRNA	Ribosomal RNA
tRNA	Transfer RNA
TYGS	Type (Strain) Genome Server
NCBI	National Center for Biotechnology Information
SRA	Sequence Read Archive
FASTQ	Sequence Read Format with Quality Scores
SPAdes	St. Petersburg genome assembler
Canu	Long-read genome assembler (Canu)
Flye	Long-read genome assembler (Flye)
Unicycler	Hybrid bacterial genome assembler
antiSMASH	Antibiotics and Secondary Metabolite Analysis Shell
LPS	Lipopolysaccharide
N50	Assembly contiguity metric
L50	Assembly length distribution metric
